# Autoencoders reloaded

**DOI:** 10.1007/s00422-022-00937-6

**Published:** 2022-06-21

**Authors:** Hervé Bourlard, Selen Hande Kabil

**Affiliations:** 1 Idiap Research Institute, Martigny, Switzerland; 2 Ecole polytechnique fédérale de Lausanne (EPFL), Lausanne, Switzerland

**Keywords:** Auto-associative multilayer perceptrons, Autoencoders, Deep neural networks, Principal component analysis, Singular value decomposition

## Abstract

In Bourlard and Kamp (Biol Cybern 59(4):291–294, 1998), it was theoretically proven that autoencoders (AE) with single hidden layer (previously called “auto-associative multilayer perceptrons”) were, in the best case, implementing singular value decomposition (SVD) Golub and Reinsch (Linear algebra, Singular value decomposition and least squares solutions, pp 134–151. Springer, 1971), equivalent to principal component analysis (PCA) Hotelling (Educ Psychol 24(6/7):417–441, 1993); Jolliffe (Principal component analysis, springer series in statistics, 2nd edn. Springer, New York ). That is, AE are able to derive the *eigenvalues* that represent the amount of variance covered by each component even with the presence of the nonlinear function (sigmoid-like, or any other nonlinear functions) present on their hidden units. Today, with the renewed interest in “deep neural networks” (DNN), multiple types of (deep) AE are being investigated as an alternative to manifold learning Cayton (Univ California San Diego Tech Rep 12(1–17):1, 2005) for conducting nonlinear feature extraction or fusion, each with its own specific (expected) properties. Many of those AE are currently being developed as powerful, nonlinear encoder–decoder models, or used to generate reduced and discriminant feature sets that are more amenable to different modeling and classification tasks. In this paper, we start by recalling and further clarifying the main conclusions of Bourlard and Kamp (Biol Cybern 59(4):291–294, 1998), supporting them by extensive empirical evidences, which were not possible to be provided previously (in 1988), due to the dataset and processing limitations. Upon full understanding of the underlying mechanisms, we show that it remains hard (although feasible) to go beyond the state-of-the-art PCA/SVD techniques for auto-association. Finally, we present a brief overview on different autoencoder models that are mainly in use today and discuss their rationale, relations and application areas.

## Introduction

Autoencoders (AE), previously called “auto-associative multilayer perceptrons”, are neural networks whose goal is to reconstruct *d*-dimensional input (i.e., observation) vectors as *d*-dimensional output vectors. AE consist of two main parts: encoder and decoder. When *d*-dimensional input is passed through the encoder, code (i.e., encoding, embedding) is extracted on the latent encoding layer. This code is then fed to the decoder to create the reconstruction for the original input at the output layer of the autoencoder. In general, AE aim to minimize the mean square error (MSE) between the original input and its reconstruction.

**Fig. 1:**
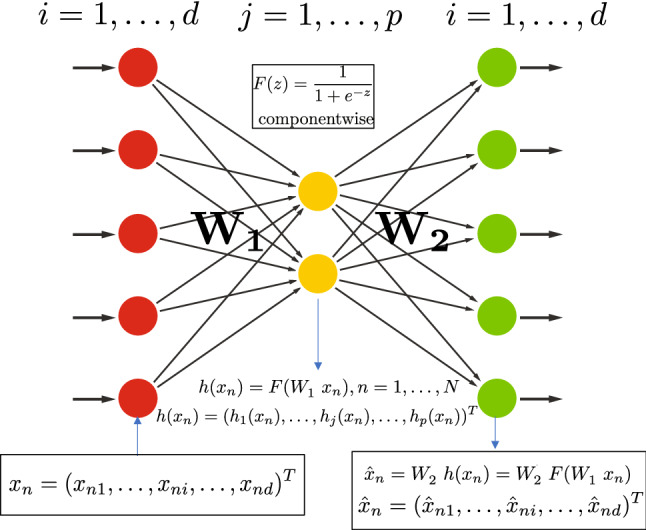
Shallow undercomplete autoencoder

In the mid-80’s, the single-hidden layered neural networks (Fig. 1) were in use as effective encoder–decoder architecture for auto-association. These models were later used as unsupervised bottleneck feature extraction models, where the code (with vector of dimension $p<d$) was considered as potentially more robust feature, as a result of a nonlinear transformation that maps a set of input observations into a new coordinate system which is more robust to the variability (e.g., providing noise reduction).

In Bourlard and Kamp (1988), it was shown that a shallow undercomplete autoencoder (i.e., autoencoder with only one fully connected hidden layer), with linear output activation function and MSE cost function, learns the weights that span the same subspace as the one spanned by the principal component vectors. Although often subject to misperceptions, or argumentation from the neural network community, it was also shown that even with nonlinearities in the hidden layer, an autoencoder trained (with the usual error back-propagation (EBP) algorithm Rumelhart et al. 1985) to minimize MSE is equivalent to linear PCA, usually explained via an eigen-decomposition of the covariance matrix or, alternatively and equivalently, performed via singular value decomposition (SVD) of the input data matrix.

Over the last 20 years, autoencoders, particularly shallow autoencoders (Fig. 1), have been widely studied and used in the context of deep learning as possibly effective unsupervised learning model to perform sophisticated nonlinear (en)coding and/or robust feature extraction (Refinetti and Goldt 2022; Baldi 2012). This resulted in different architectures discussed in this paper, including deep undercomplete autoencoders (Fig. 6), or deep undercomplete autoencoders with space expansion (Fig. 8), then reminiscent of space expansion used in support vector machines (SVM) (Ben-Hur et al. 2002) aiming at constructing a hyperplane or set of hyperplanes in a high-dimensional space, which can be later used for (linear) classification or regression.

With the recent renewed interest in deep neural networks (DNN), mainly driven by the availability of large amounts of data across multiple disciplines associated with significantly increased memory and computation resources, DNN applications have expanded significantly from image processing to speech recognition, natural language processing, time-series forecasting and neuroscience studies. In this context, autoencoders are now also widely explored as a potentially strong nonlinear feature extraction model. Besides this (unsupervised) nonlinear features extraction, there exists other notably successful use of unsupervised deep learning related to the autoencoders, including embedddings (Mikolov et al. 2013), a method used to represent discrete variables as continuous vectors, and linear/nonlinear transformers, mostly in use for speech and natural language field (Wolf et al. 2020). In addition, with this renewed interest, the growing exchange between deep learning and neuroscience (overlapped under the field of cybernetics Ashby 1961; Wiener 1948) results in the design of biologically inspired autoencoder forms in the computational neuroscience studies, especially for understanding plasticity forms and functions (Brea and Gerstner 2016; Fukai et al. 2021; Magee and Grienberger 2020).

Following the Editor’s invitation,^1^ this paper will recall some main properties of shallow autoencoders, as initially presented in Bourlard and Kamp (1988), and their equivalence to singular value decomposition (SVD) (Golub and Reinsch 1971), and principal component analysis (PCA), deriving *eigenvalues* that represent the amount of variance accounted for by each component (Hotelling 1933; Jolliffe 1986), even in the presence of their nonlinear function (sigmoid-like, or any other nonlinear functions) on their hidden units.

It is important to note that in the present paper, we do not discuss bio-inspired autoencoder architectures. Some of those architectures assume (weak) lateral connections, which is a well-established biological feature, or adopt other non-orthogonal approaches. Unfortunately, adding lateral connections within layers makes our principled mathematical developments presented here unfeasible and probably obsolete. However, we note that we will show autoencoder solutions that are not constrained to form an orthonormal basis; although, the optimal solution in the case of a MSE cost is given by (orthogonal) PCA/SVD. Of course, changing the cost function (if possible and meaningful) could also yield different non-orthogonal bases. Finally, while lateral connections are probably interesting from a biological point of view, they require more delicate mathematical formulation. We note that sparse autoencoders (Sect. 6.3) may result in similar effects since enforcing sparsity, hence constraints across hidden unit activations.

After presenting some historical context and background, we recall some main properties from Bourlard and Kamp (1988) in Sect. 3 followed by experimental analysis in Sect. 4. After validating the main properties in Bourlard and Kamp (1988) with experimental findings, we provide a brief overview of autoencoders (since 1988), including discrete input autoencoders (Sect. 5), shallow overcomplete autoencoders with special emphasis on regularized autoencoders (Sect. 6) and their connection with dictionary learning (Mairal et al. 2009) (Sect. 7), deep autoencoders underlining the importance of depth, layer-width and composition of nonlinear functions (Sect. 8), and domain-specific autoencoders which are organized based on the application domain (Sect. 9). Finally, we conclude in Sect. 10.

## Context

### Historical perspective

This work is based upon (Bourlard and Kamp 1988) which was published in the “Biological Cybernetics” journal for a couple of reasons. First, it is important to recall here that at that time there simply were no journals specifically dedicated to (artificial) neural networks. In addition, as stated by the journal’s editorial,^2^ we indeed believe that it is often important to publish articles of potentially large, multi-disciplinary interest in more generic journals like Biological Cybernetics, instead of dedicated journals (in our case to neural networks) encouraging performance, incremental improvements, in comparison with “state-of-the-art” systems on specific reference tasks. The growing number of citations to Bourlard and Kamp (1988) over the last 30+ years demonstrates the relevance of this choice.

Between 1988 and today, a few changes occurred in terms of terminology, including:

- Multilayer perceptrons (MLP) are now called *deep neural networks* (DNN), covering a large set of possible feedforward and recurrent architectures, as well as different types of processing (e.g., convolutions), and nonlinear functions (e.g., ReLU, max-pooling, etc.), making theoretical formalization much more challenging.
- Auto-associative multilayer perceptrons are now called *autoencoders*(AE), and are being used either as (en)coder channels, or as nonlinear feature extractors (e.g., nonlinear PCA/SVD) for more robust modeling.
- According to the model configuration, autoencoders are now called *undercomplete*, if the encoding layer has lower dimensionality than the input, *overcomplete*, if the encoding layer has the same or more units than the input, *shallow*, if there exist only three layers (i.e., consisting of input, encoding and output layers), and *deep*, if there exist more than three layers in the model configuration.
- A multilayer perceptron with only one hidden bottleneck layer (as commonly used in 1988) is thus now called a *shallow undercomplete autoencoder*.

In this paper, we will adopt the most recent terminology, and will further analyze (Bourlard and Kamp 1988) in the context of large datasets (not available in 1988) with modern processing resources, and different architectures of “deep neural networks” (DNN) used as “autoencoders” (AE).

### Linear algebra basics

When considering autoencoders, many useful concepts and facts about linear algebra, linear transformations, matrix rank, and sequences of linear/nonlinear transformations need to be kept in mind (Horn and Johnson 2013) and are briefly recalled here:

1. The rank of a (input) matrix *X* (input vector space) is the dimension of the vector space generated (or spanned) by its columns. This corresponds to the maximal number of linearly independent columns of *X*. Of course, for a $(N \times d)-X$ matrix, of *N* vectors of a *d*-dimensional vector space, and $N>d$, the *maximum* rank of *X* is equal to *d*.
2. A linear transformation/projection *W* of vector space *X* can only decrease its vector space rank (i.e., subspace dimension), which can *never* be increased back by any linear transformation. Only specific nonlinear functions (as discussed later) will have the potential to re-increase the rank of the matrix.
3. There is always a single linear transformation equivalence of a sequence of linear transformations, resulting in a final rank (reduction) equal (at best) to the lowest rank of the transformations in the sequence of mappings. So, keeping this in mind, it is clear that a “deep” neural network with multiple layers of linear transformations will always be equivalent to a single linear transformation (with rank constrained by the lowest transformation rank).
4. In a sequence of transformations, only nonlinear transformations can re-increase the rank of the resulting matrix, although this rank increase may be limited by the shape of the nonlinearities (Bourlard and Kamp 1988; Refinetti and Goldt 2022).
5. An intersection of subspaces is always a subspace; a union of subspaces need not be a subspace.

## Shallow undercomplete autoencoders

### Generic architecture

In this section, without getting into too much mathematical details, we recall the main conclusions from Bourlard and Kamp (1988), enriched by additional experimental evidences while also discussing some of the main misinterpretations of those results (without specific references for obvious reasons).

*Encoding* In a “classical” shallow, undercomplete, autoencoder, as illustrated in Fig. 1, a large set of *N* *d*-dimensional input vectors $x_n$

$$\begin{aligned} x_n = (x_{n1}, \ldots , x_{ni}, \ldots , x_{nd})^T \end{aligned}$$

where *T* denotes the transpose operation, is presented as training data at the input of the autoencoder, If *N* is the number of training vectors $x_n$, the entire training data can be represented by a $(d \times N)$-matrix *X*

$$\begin{aligned} X = \left[ x_1 , \ldots , x_n , \ldots , x_N \right] \end{aligned}$$

Each input vector is processed by a linear (encoding) transformation $(p \times d)$-matrix $W_1$ (parametric weight matrix for encoder)

$$\begin{aligned} W_1 = \left[ W_1 (j,i) \right] , ~ \mathrm{with} ~ j=1, \ldots , p, ~ \mathrm{and} ~i=1, \ldots , d \end{aligned}$$

resulting in:

$$\begin{aligned} y(x_n ) = W_1 x_n = ( y_1 (x_n ), \ldots , y_j (x_n ), \ldots , y_p (x_n ))^T \end{aligned}$$

The output of this linear transformation is followed by a nonlinear function *F* applied component-wise to $y(x_n )$ resulting in a $(p \times N)$-matrix *H*, composed of *p*-dimensional “hidden” vectors (also referred to as “features” or “latent variables”):

1 $$\begin{aligned} \begin{aligned} h(x_n )&= F(W_1 x_n ) \\&= (h_1 (x_n ), \ldots , h_j (x_n ), \ldots , h_p (x_n ))^T \end{aligned} \end{aligned}$$

with $p<d$, and $d \ll N$. In the case of *F* being a sigmoid function applied component-wise, we have:

2 $$\begin{aligned} h_j (x_n ) = \frac{1}{ 1+e^{- \sum _{i=0}^{d} W_1 (j,i) x_{ni}}}, \mathrm{with} ~ j=1, \ldots , p \end{aligned}$$

resulting in the hidden (latent) $(p \times N)$-matrix *H*:

$$\begin{aligned} H = \left[ h(x_1 ) , \ldots , h(x_n ) , \ldots , h(x_N ) \right] \end{aligned}$$

of maximum rank equal to *p*.

*Biases and mean normalization* For the sake of clarity, we explicitly remove the “biases” for the notation used in this paper. Instead, as typically done in literature, we extend the input vectors $x_n$ and hidden vectors $h(x_n )$ by extra $(d+1)-th$ and $(p+1)-th$ components, to have extra hidden and output weights (called biases). In Bourlard and Kamp (1988), it was shown that those biases were simply resulting a mean normalization of the *X* and *H* matrices. In the sequel of this paper, we will thus assume that *X* and *H* represent the mean-normalized matrices.

*Decoding* On the “decoder” side, an estimated reconstruction of $x_n$ is obtained by projecting back the hidden vectors $h(x_n )$ into a *d*-dimensional output space through a (hidden-to-output) linear transformation $(d \times p)$-matrix $W_2$ (parametric weight matrix for decoder). As discussed further in the paper, for real input vectors, we usually do not have a nonlinearity at the output layer, whereas in the case of discrete/binary inputs, nonlinearity at the output can exist.

At the output of the autoencoder, each input vector $x_n$ thus results in a reconstructed version ${\widehat{x}}_n$:

3 $$\begin{aligned} {\widehat{x}}_n = g(x_n ) = F(W_2 h(x_n )) \end{aligned}$$

and, considering all ${\widehat{x}}_n$, $n=1, \ldots , N$, the output matrix

$$\begin{aligned} {\widehat{X}} = \left[ g(x_1 ), \ldots , g(x_n ), \ldots g(x_N ) \right] \end{aligned}$$

*Reconstructed output* Recalling our discussion in Sect. 2.2, we know that the *maximum* rank of the resulting matrix ${\widehat{X}}$ is equal to *p*, and when using a mean square cost function the best rank-*p* approximation of *X* is given by PCA (or equivalently by SVD decomposition of the matrix). Furthermore, as recalled below, we can show that this *optimal* SVD solution can be reached by the autoencoder (via EBP training) in spite of the presence of a nonlinear function on the hidden units, here simply playing the role of a handicap instead of a potential advantage.

### Training and optimal (PCA/SVD) solution

During training, autoencoders are usually trained by minimizing a Mean Square Error (MSE) cost function, i.e., minimizing the reconstruction error between all the input vectors $x_n$ and their (en)coded/decoded (reconstructed) version ${\widehat{x}}_n = g(x_n )$, hence minimizing:

4 $$\begin{aligned} E = \parallel X - {\widehat{X}} \parallel ^{2} = \parallel X - W_2 H \parallel ^{2} \end{aligned}$$

where $\parallel \cdot \parallel$ denotes the Euclidean matrix-norm (or Frobenius norm). The training problem is to minimize *E* with respect to the parameter set $W_{1}$ and $W_{2}$.

In view of the fact that $W_{2}$ normally has rank $p < d$, (4) shows that the product $W_{2}H$ minimizing *E* is the best rank *p* approximation of *X* *in Euclidean norm*. This is a very standard linear algebra problem which can be solved as a PCA or SVD problem (Golub and Van Loan 1983; Stewart 1973), where the SVD decomposition of *X* is given by:

5 $$\begin{aligned} X = U_{n} \, \Sigma _{n} \, V_{n}^{T} \end{aligned}$$

where $U_{n}(V_{n})$ is an $d \times n$ ($N \times n$) matrix formed by the normalized eigenvectors of $X X^{T}$ ($X^{T}X$) associated with the eigenvalues $\lambda _{1} \ge \lambda _{2} \ge \cdots \ge \lambda _{n}$ and where $\Sigma _{n}$= diag[$\sigma _{1}, \sigma _{2}, \ldots , \sigma _{n}$] is a diagonal matrix with $\sigma _{i} = \sqrt{\lambda _{i}}$. For simplicity, we will assume that *X* has full row rank ($\sigma _{n} >0$). It is known (Golub and Van Loan 1983; Stewart 1973) that the best rank *p* approximation of *X* is given by

6 $$\begin{aligned} {\widehat{W}}_{2} \, {\widehat{H}} = U_{p} \, \Sigma _{p} \, V_{p}^{T} \end{aligned}$$

with $\Sigma _{p}$ = diag [$\sigma _{1}, \sigma _{2}, \ldots , \sigma _{p}$] and where $U_{p}(V_{p})$ is formed by the first *p* columns in $U_{n}(V_{n})$. Therefore, the optimal value of *H* and $W_2$ is, respectively, given by:

7 $$\begin{aligned} {\widehat{H}} = S \, \Sigma _{p} \, V_{p}^{T} \end{aligned}$$

and

8 $$\begin{aligned} {\widehat{W}}_{2} = U_{p} \, S^{-1}\, \end{aligned}$$

where *S* is an arbitrary non-singular $p \times p$ matrix which will subsequently play an important role as a *scaling matrix* in the case of nonlinearities on the hidden layer, just to make sure that the optimal (linear) SVD solution can be reached within the autoencoder and its $W_1$ and $W_2$ matrices, by positioning itself in the linear regime of the nonlinearity. The *optimal* encoder matrix $W_1$ is then just the transpose of the decoder matrix $W_2$. i.e.:

9 $$\begin{aligned} {\widehat{W}}_{1} = {\widehat{W}}_{2}^T \end{aligned}$$

*In the case of a nonlinear function F on the hidden units*, we have shown in Bourlard and Kamp (1988) that we do not need strong assumptions about the particular form of this function except that, for small values of its argument, it can be approximated as closely as desired by the linear part of its power series expansion, i.e.,

10 $$\begin{aligned} F(x) \sim \alpha _{0} + \alpha _{1}x \qquad \mathrm{for} \; x \; \mathrm{small} \end{aligned}$$

with nonzero $\alpha _{1}$. For the sigmoid, *F*(*z*) = $1/(1 + e^{-z})$, this simply gives $\alpha _{0} = 1/2$ and $\alpha _{1} = 1/4$. Based on this, we have shown in Bourlard and Kamp (1988) that, within minor modifications, the optimal values obtained above still produce the expression for ${\widehat{H}}$ required by (8). Optimal encoder weights will thus simply have to be adapted to ensure that the argument values for the nonlinear function *F* are small enough to be operating in the (optimal) linear regime. Indeed, this is the only optimal solution that will always guarantee optimum minimization of the MSE cost function.

However, it may be possible (as reported in several papers) that non-optimal MSE solutions, corresponding to local minima or to constrained/regularized (sub-optimal MSE) solutions are resulting in better “features” (i.e., hidden layer activations, encodings, embeddings), since it is well known that MSE criterion is not necessarily related to classification or regression performance. *This important observation/property will be reproduced and further discussed below.*

In the context of this particular (shallow undercomplete) autoencoder topology, when MSE is concerned, SVD will thus always yield the optimal solution. Different autoencoder topologies will also be analyzed here to get a better feeling of their potential with respect to PCA/SVD.

## Experimental results

### Initial experiments

Given the limited size of available data, CPU, and memory resources in 1988, the theory in Bourlard and Kamp (1988) (discussed in Sect. 3) could only be validated on a very limited (speech) dataset. The training database was thus composed of 60 vectors in ${{\mathcal {R}}}^{16}$ (hence *X* is a $16 \times 60$ real matrix). These were cepstral vectors obtained from 10-ms frames of speech signal and corresponded to the mean vectors associated with the states of phonemic hidden Markov models Bourlard et al. (1985). We determined the optimal weight matrices ${\widehat{W}}_{1}$, ${\widehat{W}}_{2}$ for a rank 5 approximation (corresponding to 5 hidden units) by the SVD of *X* (as described in Sect. 3) in Bourlard and Kamp (1988). Then, we used these weight matrices to initialize the autoencoder before the initialization of the error-backpropagation (EBP) (Rumelhart et al. 1985) training algorithm. We observed that the EBP was unable to improve the weight parameters by reducing the MSE cost function any further. Moreover, when starting the EBP training algorithm several times with random weights, it always got stuck in diverse local minima, yielding higher error values.

### Larger datasets

Today, we obviously have access to larger and more challenging datasets with nearly unlimited computation and memory resources. Thus, we made additional experiments on a commonly used, moderate size handwritten digits dataset (MNIST) and a challenging speech dataset (AMI) with more complex patterns.

#### MNIST handwritten digits dataset

The MNIST (Modified National Institute of Standards and Technology database) dataset^3^ consists of 60,000 training and 10,000 testing images of $28 \times 28$ pixels grayscale images of handwritten digits between 0 and 9. The dataset is well-mastered for benchmarking purposes and mainly used for digit classification task. The state-of-the-art models (usually with convolution layers) achieve a classification accuracy above 99%.^4^

We split MNIST train set into the subsets of 50,000 and 10,000 to use as training and validation sets, respectively. As our autoencoders solely consist of fully connected layers, we flattened 28x28 images into 784-dimensional vectors. As for preprocessing, the image pixel values are set to a [0–1] range.

Based on the L-curve method (Hansen and O’Leary 1993), we picked $p=50$ as the “optimal” number of hidden units, as well as the number of PCA eigenvectors. Autoencoders were initialized either by Pytorch default initialization or PCA initialization (i.e., dumping the eigenvectors obtained with PCA/SVD as decoder weights ${\widehat{W}}_{2}$, as given by (8) and its transpose as encoder weights, ${\widehat{W}}_{1} = {\widehat{W}}_{2}^T$, as given by (9)). Each autoencoder model was trained with a learning rate of 0.0001, weight decay of 0.0001, batch size of 100 with Adam optimizer. To avoid overfitting, we checked MSE on validation set during training. Finally, for further cross-validation purposes, the neural network trainings were always performed with three different seeds.

In Table 1, we compared MSEs from the autoencoders with different parameter initialization schemes (e.g., Pytorch default, PCA initialization) and linear or nonlinear (e.g., sigmoid) hidden units. Table 1 validates the theory presented in Sect. 3 and indicates that MSE difference between different architectures is not significant.

*MSE vs accuracy* Although the aforementioned conclusions about autoencoders and PCA equivalence clearly holds, it is also known (and actually observed in practice, depending on the complexity of the data) that lower MSE does not automatically imply “optimal” features, yielding the best classification performance. To further validate our work, we decided to send the resulting latent features (i.e., codes, encodings, hidden unit activations) into a reasonably simple classifier.

**Table 1:** Reconstruction and digit classification performance (in terms of MSE and accuracy, respectively) on MNIST with mere PCA with 50 principal components (first row), shallow undercomplete autoencoders with different parameter initialization schemes (i.e., Pytorch default, PCA initialization) and nonlinearity (i.e., sigmoid) on hidden layer. Results are in line with the theory presented in Sect. 3 as the reconstruction performances (wrt MSE) between different architectures is not significant

Initialization	Hidden	MSE	Accuracy (%)
PCA	linear	9.14	$98\pm 0$
Pytorch default	linear	9.01	$98\pm 0$
PCA initialization	sigmoid	9.11	$97\pm 0.5$
Pytorch default	sigmoid	9.11	$97\pm 0.5$
Baseline	No AE/PCA		$96 \pm 0$

For MNIST, the classifier consisted of 50 inputs units (as number of hidden units in the autoencoder, $p=50$), one nonlinear expansion layer (1200 units with sigmoid activation) followed by a softmax output layer (10 units representing 10 digits in MNIST). The classifier was trained with cross-entropy (CE) criterion and learning rate 0.01 for 20 epochs. The baseline digit classification accuracy directly using the input features (without PCA or autoencoder) was of 96%. Again, from Table 1, we see that subspace reduction helps accuracy, although no significant difference can be observed from different configurations.

#### AMI meeting corpus

The AMI (Augmented Multiparty Interaction) dataset^5^ is a multi-modal dataset consisting of 100 hours of meeting recordings. For our experiments, we used IHM (Independent Head-mounted Microphones) portion of the AMI Meeting Corpus with 81 hours of train, 9 hours of development, and 9 hours of evaluation data.

The input features had the dimension of 351 (39-dimensional Mel-Frequency Cepstral Coefficients (MFCC) + $\Delta$ + $\Delta \Delta$ x 9 frames of temporal context). The number of phone classes was equal to 176. For preprocessing, the acoustic features were centered. Each model was trained with learning rate 0.0001, weight decay 0.0001, batch size 100 with Adam optimizer, and early stopping procedure for maximum of 150 epochs. Initialization and optimal bottleneck dimension of the autoencoders were handled by following the same methodology for the MNIST. L-curve method Hansen and O’Leary (1993) resulted in “optimal” performance for a latent space of dimension $p=250$.

After autoencoder training, the encodings (i.e., hidden layer activations or PCA output) were normalized (zero mean, unit variance) and passed to a simple classifier. The same classifier configuration as MNIST is trained with learning rate 0.01 for 50 epochs. The reference phone labels (i.e., true alignments) for the classifier training were extracted using a state-of-the-art recognition model in Kaldi toolkit (Povey et al. 2011). The AMI IHM phone classifier thus consisted of 250 units at the input layer (as $p=250$ autoencoder hidden units), one nonlinear expansion layer (with 1200 units and sigmoid activation) followed by a softmax output layer with the number of units equal to the number of classes in the corresponding task (176 phone classes for AMI IHM), and trained along a Cross-Entropy (CE) criterion. The baseline accuracy is 53%.

**Table 2:** Reconstruction and phone classification performance (in terms of MSE and accuracy respectively) on AMI IHM with mere PCA with 250 principle components (first row), shallow undercomplete autoencoders with different parameter initialization schemes (i.e., Pytorch default, PCA initialization) and nonlinearity on hidden layer. PCA obtains significantly lower MSE and slightly better frame-based phone classification accuracy

Initialization	Hidden	MSE	Accuracy (%)
PCA	linear	0.0015	$57\pm 0$
Pytorch default	linear	0.005	$56\pm 0.6$
PCA initialization	sigmoid	0.05	$55\pm 0$
Pytorch default	sigmoid	0.05	$55\pm 0.5$
Baseline	No AE/PCA		$53 \pm 0$

### Discussion and potential advantages of AE

In spite of the fact that shallow undercomplete autoencoders are equivalent to PCA/SVD, they may still be exploited for several of their potential advantages, briefly discussed below.

*Iterative, batch-based, online, error back-propagation training* One could object that the autoencoders (shallow, or deep, as discussed later) and their associated EBP training algorithm allow for online learning. It is indeed true that for very large *N*, hence very large datasets, it may become difficult to perform exact PCA or SVD decomposition, given possible algebraic singularities or instabilities. EBP may thus provide an important advantage when the number of training data becomes very large (as often the case today). Batch-based, stochastic, back-propagation applied to autoencoders can then be used to estimate PCA/SVD (en)coding/decoding matrix transformation, although there is also an online version of standard, algebraic PCA (Bunch and Nielsen 1978).

*Parallel processing* Similarly, while autoencoder training can be implemented on fast parallel hardware, similar mappings can be made for SVD. Perhaps the only hardware-oriented argument that may favor autoencoders is that their training can be done with lower precision (e.g., 16 bits for weights and 8 bits for activation), while SVD usually requires more precision (typically 32-64 bit floating point is used).

*Online adaptation* In the same spirit, autoencoders can easily be adapted to new data, to be continually enriched and refined with additional and updated data, although PCA adaptation also exists (VJ et al. 1984).

*Cross-validation* One popular and convenient technique to avoid overtraining in deep neural networks, including autoencoders, is to perform cross-validation on an independent validation set and early-stop training when performance on the this set starts to decrease (Krzanowski 1987; Morgan and Bourlard 1990). While this is a very efficient and well understood approach towards robust DNN training, there is usually no strong equivalent in linear algebra.

*Regularization* In addition to cross-validation (or as an alternative to it), multiple regularization schemes have also been developed and adapted to different DNN architectures, as further discussed in Sect. 6.2. Those regularization methods are usually very efficient and well understood, however, costly to implement in advanced linear algebra (Lu et al. 2016).

*Using different cost functions* DNN training usually based on variants of the EBP algorithms can easily accommodate complex models, as well as multiple cost functions, as long as those cost functions are differentiable and can be casted into the chain rule of differentiation. This is also a specific, well-mastered advantage of DNNs, although modified PCA/SVD approaches based on different cost functions also exist (Guo et al. 2004; He et al. 2011).

*Dealing with discrete/binary data* As discussed in detail in Sect. 5, DNNs, autoencoders in particular, are agnostic to the properties of their inputs and can treat real or symbolic inputs indifferently, as well as a mix variable types. Of course, linear algebra, and standard PCA/SVD approaches are really not well adapted to that type of problem ( e.g., since it doesn’t make any sense to do PCA on binary data, or poorly defined data distributions) (Schein et al. 2003; De Leeuw 2006).

*Further discussions* More strict mathematical treatments about the absence of local minima, the presence of saddle points, learning properties, and relationships with principal component analysis are possible (Baldi and Hornik 1989, 1995).

## Discrete input autoencoders

### Encoding and decoding of discrete inputs

As discussed previously, there is (usually) no nonlinear function at the output layer for autoencoders with continuous input/output vectors. However, if the input/output vectors are binary, then logistic-like functions are needed at the output. In that case, of course, the discussions about the relationships between shallow undercomplete autoencoders and PCA/SVD have to be revisited in the case of discrete (binary) input/output networks since:

- PCA/SVD on discrete, symbolic or binary valued vector spaces does not make sense
- While this is possible with autoencoders, the results may yield fallacious interpretations and conclusions. For example, there have been many papers illustrating the magic power of autoencoders by experimentally showing that discrete autoencoders have the ability to perfectly reconstruct infinitely long binary input with only one single hidden layer of two nonlinear (sigmoid) units; hence, “encoding” infinitely long binary vectors into a 2-dimensional (real valued) vector. However, of course, there is no magic here since it is always possible to encode arbitrarily long binary input into an infinite precision real-valued vector from which we can reconstruct the binary input (providing nonlinear gating/rounding).

However, when used appropriately, adding additional constraints, or proper regularization (e.g., avoiding infinite precision) this binary autoencoding can be used to map a discrete space into a continuous space (then called embedding) possibly exhibiting interesting topological properties, as further analyzed and discussed below. Notably successful use of deep learning, related to autoencoder embeddings, a common method now used to represent discrete variables as continuous vectors, has found practical applications with word embeddings for machine translation and entity embeddings for categorical variables (Levy and Goldberg 2014).

### Discrete embeddings

**Fig. 2:**
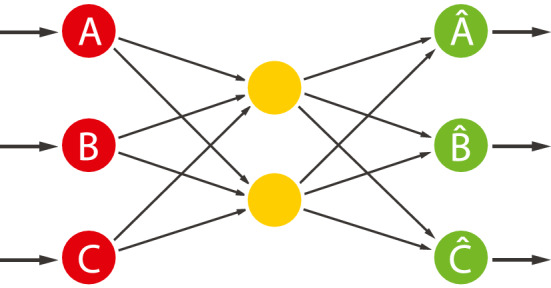
Shallow undercomplete on discrete variables

Let us consider the autoencoder embedding example illustrated in Fig. 2, where we train the system to embed sequences of $\{ A, B, C \}$ statistical symbols to be reconstructed at the AE output to minimize a given cost function (e.g., MSE, CE etc.). Ideally, the resulting discrete shallow autoencoder should model the joint probability distribution *P*(*A*, *B*, *C*) on its hidden layer, given some regularization constraints further discussed below.

In this case, the role of the nonlinear function *F* is usually different for the hidden layer(s) and the output layer. On the hidden units, nonlinearities are necessary to generate nonlinear representations of the input. Indeed, as recalled in Sect. 2.2, a sequence of linear transformations is always equivalent to a single linear transformation, possibly with an input matrix rank reduction in the case of a “bottleneck”. In the case of discrete inputs (often turned into binary representations), it can also be shown that a sigmoid function will, in theory, generate all possible high order moments of the input. However, its form is certainly not restricted to a saturating nonlinearity such as the sigmoid function. A sinusoidal function could also be used or, as is usually done with radial basis functions, a quadratic function might be used, in which case we generate only second-order moments on the hidden units.

Recalling forward (1) and (2) for the autoencoders, we thus have:

11 $$\begin{aligned} h(x_n ) = F( {W}_{1} {x}_{n}) \end{aligned}$$

where *F* is a nonlinear function, typically a sigmoid function, that is applied to each component of its argument, and where $x_n$ are now possible stochastic sequences of the discrete *A*, *B*, *C* symbols in binary forms. The reconstructed version of the *A*, *B*, *C* sequences is then given by:

12 $$\begin{aligned} g(x_n ) = F( {W}_{2} {h}(x_n )) \end{aligned}$$

where *F* has to be a logistic function to reconstruct binary outputs.

As done previously, let $$x_{ni}, \, i=0,\ldots ,d$$, denote the components of the input (now binary) vector $$ {x}_{n}$$. When using a sigmoid function on the hidden units, the *j*-th component of $$h(x_n )$$ is then expressed as (2):

13 $$\begin{aligned} h_{j}(x_n ) = \frac{1}{ 1+e^{- \sum _{i=0}^{d} w_{ji} x_{ni} }} \end{aligned}$$

where $W_1 (j,i)$ of (2) is here simply written as $w_{ji}$ for the sake of simplicity; where $w_{ji}$ represents the weight between *j*-th input unit and the *i*-th hidden unit.

In the case of *F* being a sigmoid function (as an example), and using its Taylor expansion, we get:

14 $$\begin{aligned} h_{j}(x_n ) = \sum _{k=0}^{\infty } \alpha _{k} \left( \sum _{j=0}^{d} w_{ji} x_{nj}\right) ^k \end{aligned}$$

where $\alpha _{k}$ are the Taylor coefficients.

In the case of binary inputs, we have $x_{nj}^{k} = x_{nj}, \, \forall k>0$, and (14) can then be rewritten as:

15 $$\begin{aligned} h_{j}(x_n )= & {} \alpha _{0} + \sum _{i=0}^{d} \alpha _{i} x_{ni} + \sum _{i \ne k}^{d} \alpha _{ik} x_{ni} x_{nk} \nonumber \\&+ \sum _{i \ne k \ne \ell }^{d} \alpha _{ik \ell } x_{ni} x_{nk} x_{n \ell } + \ldots \end{aligned}$$

Thus, for each component of $$h_j (x_n )$$, the nonlinear function *F* generates a *linear combination* of all $$2^{d}$$ possible cross-products of the *d* binary inputs (i.e., pairs, triplets, $$\ldots , d$$-tuples). The coefficient $$\alpha $$ of each cross-product depends on the weight matrix $$ {W}_{1}$$ which is adapted during the training so as to activate the relevant cross-products (i.e., those which are *typical of the training patterns* and *insensitive to the noise*). At the same time, $$ {W}_{2}$$ is also adapted to optimize the *reconstruction* on the basis of the generated cross-products.

In summary, discrete autoencoders can be used to:

- Map sequences of stochastic discrete symbols $\{ A, B, C \}$ into a continuous space
- Model the joint probability distribution *P*(*A*, *B*, *C*) in that continuous space
- Extract (discrete/symbolic) input correlations through multiple sets of different-order correlation/cross-products of the inputs that are optimal for reconstruction, (i.e., robustly map discrete inputs into a continuous space while preserving important relationships between symbols). For instance, in the case of document classification or translation, symbolic contextual information will be exploited to generate a continuous space where topological properties are representative of that information.

### Most common embeddings

Binary input autoencoders thus have conceptual similarities with different embedding methods used today (Gutiérrez and Keith 2018), including the popular *Word2Vec* technique (Mikolov et al. 2013), which is practically in use for machine translation and entity embeddings for categorical variables (Zou et al. 2013).

**Fig. 3:**
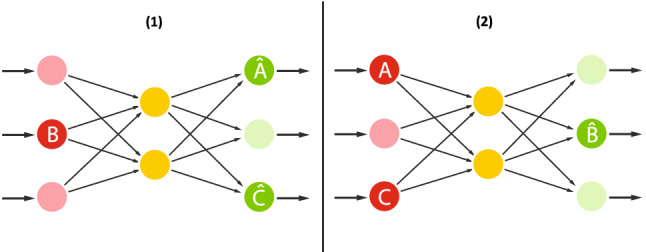
Embeddings

Word2Vec (Mikolov et al. 2013) is one of the most well-known technique for learning word embeddings. It is originally proposed as shallow, fully connected neural networks, resembling shallow undercomplete autoencoders (Fig. 3).The only non-linearity is the softmax calculations in the output layer. Similar to the autoencoders, depth and neural building blocks (e.g., fully connected layers, convolutional layers, recurrent layers) of the architecture can be changed.

In Word2Vec technique, the input data in one-hot encoding^6^ format is subjected to a linear information bottleneck to learn continuous, dense latent representation. This is also similar to the matrix factorization; however, the learning objective is different. With the softmax (classifier) at the output layer, it uses cross-entropy as loss function. It learns by back-propagating the gradient from the softmax output to the dense word representations in the hidden bottleneck layer so that the cross-entropy loss of the softmax classifier is minimized.

Today, word embeddings are obtained using two different Word2Vec approaches; skipgram and CBOW, still aiming at optimally modeling *P*(*A*, *B*, *C*), but through two different regularization constraints and different priors *P*(*A*, *C*) or *P*(*B*).

In *skipgram*, illustrated in Fig. 3(1), the goal is to predict the (target) contextual words $\{ {\hat{A}} , {\hat{C}} \}$ given the sole source word $\{ B \}$ as input, hence modeling *P*(*A*, *C*|*B*).

In *CBOW*, illustrated in Fig. 3(2), the goal is to predict the target word ${\hat{B}}$ given the (source) context words $\{ A,C \}$.

However, in all cases, the discussion presented in Sect. 5.2, regarding the interpretation of how the continuous embeddings are being built, remains valid.

## Shallow overcomplete autoencoders

### Generic architecture

As explained in Sect. 3, undercomplete autoencoder learns to span the same subspace as PCA under certain conditions such as linear decoder (i.e., linear output layer), MSE as loss function, and real-valued input data. Having smaller code dimension than the input dimension (being undercomplete) forces the autoencoder to learn the salient features as encodings from the training data. Apart from this, of course, there exist other ways to design autoencoders.

The use of nonlinear activations in the encoder and decoder, the composition of different unit types (e.g., convolution layers, recurrent layers), the higher code dimension than the input dimension (i.e., being overcomplete), and the depth of the encoder and decoder while preserving the symmetry of the network architecture are common ways to build an autoencoder. However, it is important to keep in mind that if the autoencoder is given too much modeling power, it can simply learn the identity function (i.e., perfectly reconstructing the input data at the output layer) between the input data and output reconstruction. This phenomenon is also known as identity mapping.

**Fig. 4:**
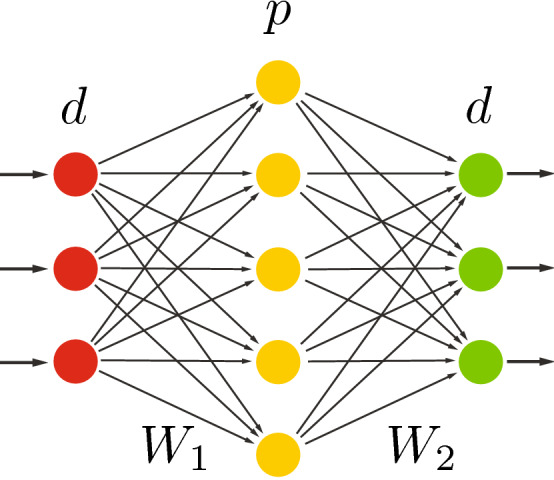
Shallow overcomplete autoencoder with (nonlinear) space expansion (number of hidden units $p>> d$). Of course, in this case, if no regularization is applied, this autoencoder will overfit and simply do identity mapping while learning meaningless encodings on the hidden layer. Different regularization strategies to avoid this phenomenon is discussed in the following sections

In this section, we focus on the shallow overcomplete autoencoders (i.e., autoencoders with only one hidden layer whose code dimension is greater than the input dimension ($p>> d$)) as illustrated in Fig. 4. Due to overcompleteness (and nonlinearity if exists), the increased modeling capacity of AE can result in identity mapping. In this case, some form of constraint/regularization (e.g., sparsity, contraction, noise) should be introduced during training to guide the autoencoder for learning meaningful encodings, as explained in the sections below.

### Modeling capacity and regularization

Modeling capacity indicates the complexity of the relationships in the data (i.e., patterns) that the model can express. The most common way to estimate the capacity of a model is to count the number of parameters. More parameters indicate higher capacity. Hence, overcompleteness increases the modeling capacity of the autoencoder.

In other words, given the input data, if AE tends to overfit, it can simply end up learning the identity function. Thus, high modeling capacity is not always desired. To avoid this trivial identity function, different forms of constraints are exploited. Autoencoders with such constraints are known as *regularized autoencoders*.

These constraints are usually in the form of additional regularizer term(s) in the AE loss function. More precisely, when the loss function for the autoencoder training (4) is rewritten as follows, with $g(x_n )$ in (3),

16 $$\begin{aligned} L_{\mathrm {AE}}=\frac{1}{N} \sum _{n=1}^{N}\left( x_{n}-g\left( x_{n}\right) \right) ^{2} \end{aligned}$$

The loss function for the regularized autoencoder can then be stated as

17 $$\begin{aligned} L_{\mathrm {RAE}}=\frac{1}{N} \sum _{n=1}^{N}\left( x_{n}-g\left( x_{n}\right) \right) ^{2} +\lambda L_{\mathrm {reg}} \end{aligned}$$

where $\lambda$ denotes the regularization weighting term, tuning the penalty term with respect to the reconstruction loss (16) for learning meaningful encodings.

These meaningful representations can be in the form of sparse codes by means of sparse autoencoders with sparsity constraint on the hidden unit activations. Alternatively, by means of denoising and contractive autoencoders, the robust representations (robust to the perturbations in the input data) can be learned in the encoder.

### Sparse autoencoders

The sparsity constraint is generally in the form of a penalty term (regularizer term) for constraining the hidden unit activations (i.e., encodings). Depending on the type of this additional penalty term in AE loss function, several sparse autoencoder (SAE) variants can be found in literature.

Sparse autoencoders with L1 norm penalty seems to be the most natural choice, and is also used in sparse coding (Olshausen and Field 2004) as further discussed in Sect. 7.

18 $$\begin{aligned} L_{\mathrm {SAE}}= L_{\mathrm {AE}} +\lambda \sum _{n=1}^{N} \Vert h(x_n ) \Vert _1 \end{aligned}$$

SAE with Kullback–Liebler (KL) divergence (Ng 2011) applies KL penalty to enforce sparsity on sigmoidal hidden unit activations (i.e., sigmoid function on the hidden layer). KL (Kullback and Leibler 1951) estimates the distance between predetermined desired average activation $\rho$ and the average activation for the hidden units. *p* in 19 denotes the number of hidden units.

19 $$\begin{aligned} L_{\mathrm {SAE}}= L_{\mathrm {AE}} +\lambda \sum _{j=1}^{p} \mathrm {KL}\left( \rho \Vert {\hat{\rho }}_{j}\right) \end{aligned}$$

The desired average activation $\rho$ is set to a very small value (e.g., 0.002). When $\hat{\rho _j}$ (average activation of hidden unit *j*) is close to $\rho$, KL penalty is expected to be low. However, in the opposite case, KL penalty grows rapidly, finally approaching to infinity when $\hat{\rho _j}$ approaches to 1.

20 $$\begin{aligned} \mathrm {KL}\left( \rho \Vert {\hat{\rho }}_{j}\right) =\rho \log \frac{\rho }{{\hat{\rho }}_{j}}+(1-\rho ) \log \frac{1-\rho }{1-{\hat{\rho }}_{j}} \end{aligned}$$

### Contractive autoencoders

Contractive autoencoder (CAE) (Rifai et al. 2011) uses an additional penalty term in the loss function (21). The penalty comprised of the squared Frobenius norm of the Jacobian matrix of the encoder.

21 $$\begin{aligned} L_{\mathrm {CAE}}= L_{\mathrm {AE}} +\lambda \sum _{n=1}^{N}\left\| J_{f}(x_{n})\right\| _{F}^{2} \end{aligned}$$

This Jacobian term promotes local invariance to displacements/alterations in many directions around the training samples so that the model gets less sensitive to the small perturbations in the input data. That is, this penalty term forces the autoencoder to extract encodings whose all dimensions are contracted. However, at the same time, the reconstruction error prevents the model from contracting the dimensions along the (true) data manifold.

### Denoising autoencoders

Imposing penalties on the hidden unit activations is not the only way to guide the autoencoder for learning meaningful encodings. Denoising autoencoder (DAE) (Vincent et al. 2008) uses stochastic corruption of the (clean) input data $x_n$ as regularizer during model training. The corruption of the input, resulting in $\tilde{x}_{n}$, can be additive isotropic Gaussian noise, salt and pepper noise for gray-scale images, and/or masking noise (i.e., setting some randomly chosen inputs to 0 independently per instance).

DAE adopts different criterion to evaluate the performance of the reconstruction (22), based on the difference between the clean data $x_n$ and the associated noisy data $\tilde{x}_{n}$:

22 $$\begin{aligned} L_{\mathrm {DAE}}=\frac{1}{N} \sum _{n=1}^{N}\left( x_{n}-g\left( \tilde{x}_{n}\right) \right) ^{2} \end{aligned}$$

During training, DAE takes partially corrupted training instances as input and tries to reconstruct the original, uncorrupted instances. In this way, the model is forced to find the true data manifold. It is important to note that after the DAE is trained, the model is used to extract meaningful higher-level representations without corrupting the input data.

### Variational autoencoders

Variational autoencoder (VAE) (Kingma and Welling 2019) replaces the deterministic functions in the autoencoder configuration by stochastic mappings. That is, the encoder does not map each instance to a single point in the embedding space, but to a distribution instead. This is usually a normal distribution, defined by its mean and standard deviation. Then, a reconstruction is produced by sampling that distribution and propagating the results through the decoder network. Since VAE allows sampling from the learned distribution, its applications usually involve generating new instances (Dosovitskiy and Brox 2016).

VAE assumes that a latent, unobserved random variable *z* exists, which by some random process leads to the observation *x*. Its objective is thus to approximate the distribution of the latent variable given the observations. The loss function of VAE can be decomposed into terms of single datapoints. For the sake of simplicity, in ( 23), the loss function is for one datapoint $x_n$.

23 $$\begin{aligned} \mathcal {L}_{\mathrm {VAE}}(\theta , \phi ; \mathbf {x_n})= & {} \mathrm {KL}\left( q_{\phi }({\mathbf {z}} \mid \mathbf {x_n}) \Vert p_{\theta }({\mathbf {z}})\right) \nonumber \\&-{\mathbb {E}}_{q_{\phi }({\mathbf {z}} \mid \mathbf {x_n})}\left[ \log p_{\theta }(\mathbf {x_n} \mid {\mathbf {z}})\right] \nonumber \\ \end{aligned}$$

where *q* is the distribution approximating the true latent distribution of *z*, and $\theta$, $\phi$ are the parameters of encoder and decoder distributions, respectively.

In ( 23), the first term acts as regularizer between the encoder’s distribution and the standard normal distribution. That is, if the latent representations *z* produced by the encoder are different than those from normal distribution $p_{\theta }({\mathbf {x}})$, this term penalize the loss. However, the second term in ( 23) is the reconstruction loss, promoting the decoder to reconstruct the data $x_n$. If the reconstruction does not comply with the input data well, it can be said that the decoder parametrizes a distribution which fails to model the true distribution of the data.

Hence, the objective function (23) combines the clustering behavior of the reconstruction loss function with a regularization loss which forces the distribution to be as similar as possible to a multivariate unit Gaussian. This helps the VAE extract a very compact encoding which only preserves the necessary information to provide a reconstruction of the input.

### Adversarial autoencoders

Similarly to VAE, adversarial autoencoder (AAE) (Makhzani et al. 2015) forces a prior distribution on the encodings, which allows to sample new instances by taking points from this space and projecting them onto the original feature space via reconstruction. However, AAE is built based on the concept of generative adversarial network (GAN) (Goodfellow et al. 2014). The model consists of a discriminative network and an autoencoder which are concurrently trained. The encoder of the autoencoder acts like the generator network. It is trained to fool the discriminator, while the discriminative network tries to distinguish the distribution samples from the codes belonging to actual training instances. In this way, the learned encodings are expected to follow the imposed distribution. Hence, the AAE is also capable of generating new meaningful instances.

## Shallow AE and dictionary learning

In this section, we show that shallow overcomplete sparse autoencoders, as discussed in Sect. 6, with linear activation, tied weights (i.e., encoder and decoder weights are transpose of each other), no bias and L1 norm regularization on the hidden unit activations (i.e., codes, encodings, embeddings) exhibit similar mathematical properties with dictionary learning (Mairal et al. 2009) and share the same goal of projecting the data to higher dimensional sparse spaces.

**Fig. 5:**
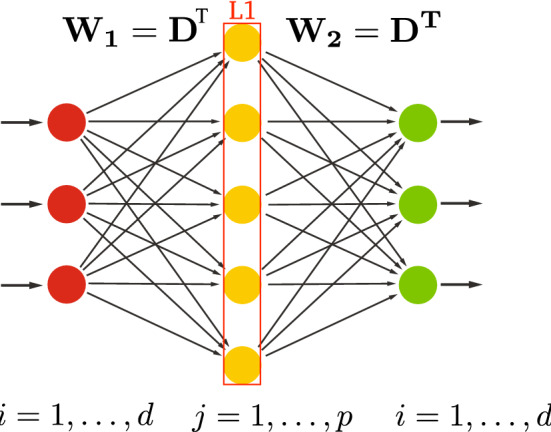
Interpretation of a shallow overcomplete sparse autoencoder in the context of dictionary learning where the coder weights $W_1$ are equivalent to the transpose of the *dictionary atoms* matrix $D^T$, and the decoder weights are the transposed of $W_1$

In shallow overcomplete sparse autoencoder illustrated in Fig. 5, input data is thus mapped to a sparse code:

24 $$\begin{aligned} h_{n} = D^T x_{n} \end{aligned}$$

where $D^T$ is the encoder weights of the autoencoder, and $h_{n}$ is the *p*-high-dimensional sparse code of $x_{n}$. The encoder weights are just the transpose of the decoder weights *D*, which is a $(p \times d)$-rectangular matrix. Hence, *D* here acts like the overcomplete dictionary in sparse coding setting.

The overcomplete projection dictionary (decoder weights) *D* is then solved by solving the following optimization problem:

25 $$\begin{aligned} \min _{D,h_{n}} \sum _{n=1}^{N} \Vert x_{n} - DD^Tx_{n} \Vert _2^2 + \lambda \Vert h_{n} \Vert _1 \end{aligned}$$

where $\lambda$ is an hyperparameter controlling the L1 norm regularization term which promotes sparsity of $h_{n}$. This is analogous to basis pursuit (Chen et al. 2001) in sparse recovery theory and to LASSO regression (Tibshirani 1996) in statistics.

While shallow overcomplete sparse autoencoder is trained to solve this optimization problem (25), forward pass can be taken as sparse coding (Olshausen and Field 2004) step in dictionary learning (learning $h_{n}$ while *D* is fixed), since we obtain high-dimensional sparse code $h_{n}$ on the hidden layer. Similarly, backward pass is analogous to the dictionary update step in dictionary learning (optimizing over *D*), as decoder weights are updated based on the distance between the original input and the reconstructed input.

## Deep autoencoder architectures

### Context and rationale

In Sect. 3, we have recalled that shallow undercomplete autoencoders will never be able (in theory) to outperform PCA/SVD, and that in the best case the autoencoder produces the best rank-*p* (with *p* equal to the number of hidden units), independently of the presence and types of nonlinear function used on the hidden layer. This theoretical claim was then further validated through experimental results reported in Sect. 4 on MNIST handwritten digits, and AMI IHM speech meeting data sets. In both cases, multiple system configurations and initialization schemes (either exploiting PCA or default Pytorch initialization) were used. Although only a very small subset of those experiments are reported in Tables 1 and 2, it was always observed that PCA/SVD was yielding the best solutions, at least in terms of the MSE cost function.

However, following the common belief, we hoped to improve these results by using deeper architectures where the power of nonlinearities, bottlenecks, or space expansion could be fully exploited. Indeed, beyond the undercomplete autoencoders discussed in Sect. 3, it is clear that nonlinear space expansion (produced through a nonlinear transformation) of the hidden layer followed by a linear undercomplete bottleneck layer provides more potential for rank reduction (explained in Sect. 2.2) and encodings for classification tasks. This is then reminiscent of the key ideas behind SVM and other kernel-based learning methods (Vapnik 1999; Cristianini and Shawe-Taylor 2000)

While designing the neural networks, the key design decisions are choosing the depth of the network and the width of each layer. Deeper networks are often able to use far fewer units per layer and generally far fewer parameters, hence frequently generalizing to the test set. In addition, depth can exponentially reduce the computational cost of representing some functions (Goodfellow et al. 2016). Different from the shallow autoencoders illustrated in Figs. 1 and 4, autoencoders can also be consisted of several hidden layers in the encoder and decoder (provided that the model symmetry is preserved). However, they can also tend to be harder to optimize. A common strategy for training a deep autoencoder is to greedily pretrain the deep architecture by training a stack of shallow autoencoders. So, we often encounter shallow autoencoders, even when the ultimate goal is to train a deep autoencoder (Bengio et al. 2007).

In the following subsections, we briefly discuss the different (3 hidden layers) architectures that have been trained to explore the potential of deeper configurations. Whenever possible, we experimented with different initialization schemes, including possible PCA/SVD solutions, as discussed below. During our experiments, we observed that (1) bottleneck dimension is crucial, (2) instead of using all linear or all nonlinear hidden layers, nonlinear expansion (layer) followed by linear compression (layer) obtains the most promising performance among deep autoencoder configurations presented below. However, as we have not managed to have any of these deep architectures yielding significantly better reconstruction and classification performance (at the same time) compared to our initial shallow undercomplete autoencoder, we will not present experimental results on MNIST or AMI IHM.

### Deep undercomplete

As explained earlier (in Sect. 2.2), the expressive power of linear features is very limited. They cannot be stacked to form deeper, more abstract representations since the composition of linear operations yields another linear operation at the end. In other words, stacking all linear layers is actually pointless. However, when stacking linear transformations, nonlinear activation functions *F*(.) can actually *increase* the rank of the matrix, theoretically resulting in better modeling potential, well aligned with the Universal Approximation Theorem (Hornik et al. 1989), which states that sequences of nonlinear transformation helps the neural networks to perform more complex tasks.

**Fig. 6:**
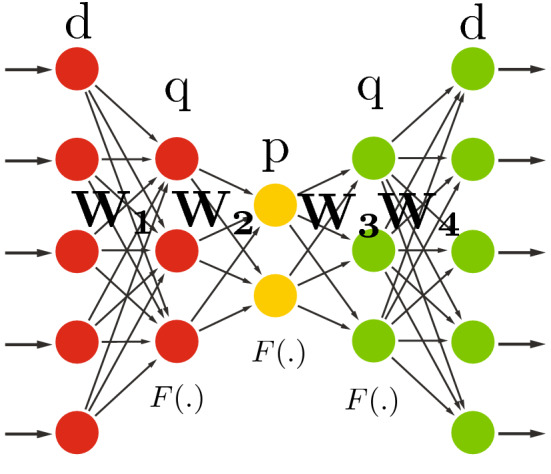
Deep undercomplete autoencoder. In this configuration, we use two nonlinear encoding layers (with nonlinear function *F*(.), usually a sigmoid) followed by similar nonlinear decoding layers, with the hope to better exploit the nonlinear transformations. Starting from *d* input units, we have $$q > p $$ as the number of units in the hidden encoding/decoding layers

We thus used the deep autoencoder illustrated in Fig. 6, with $d>q>p$. Although it is often claimed that deep autoencoders yield better compression compared to shallow or linear autoencoders (Hinton and Salakhutdinov 2006), we never managed to observe any significant improvement compared to our initial shallow undercomplete autoencoder, in terms of MSE or classification performance reported in Sect. 4. Of course, whatever we do, we also know that this autoencoder will never yield lower MSE than a shallow undercomplete autoencoder with a single hidden layer of *q* units. However, we could hope that the second layer would play a reasonable regularization role. While doing this, we also used different initialization schemes, including “ad-hoc” hierarchical PCA/SVD, i.e., first using PCA/SVD to reduce the input matrix rank to *d* and use the resulting PCA/SVD transformation as initial $W_1$ matrix (and $W_4$ as $W_1^{T}$). The generated rank-*q* data was then used as input to a PCA/SVD transformation to further reduce the rank of the data to *p*, while using the resulting transformation for the initialization of $W_2$ (and $W_3$ as $W_2^{T}$).

### Deep overcomplete

**Fig. 7:**
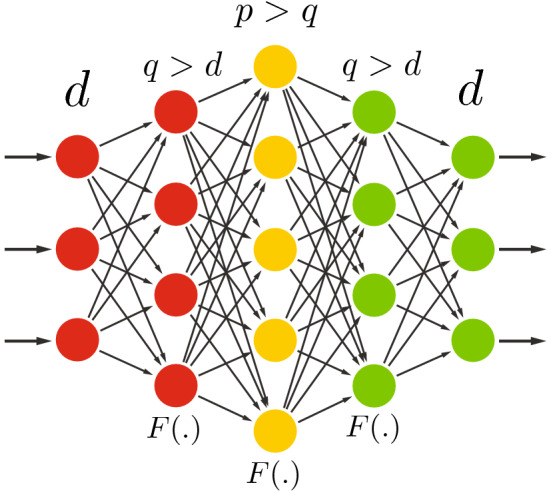
Deep overcomplete autoencoder. The increase in number of model parameters and the composition of nonlinearities on the hidden layers boost the modeling capacity. Hence, the regularization techniques (Sect. 6) can be introduced to deep overcomplete autoencoders for avoiding identity mapping

As explained in Sect. 6, and illustrated in Fig. 7, going overcomplete increases the number of hidden units in the fully connected layers; hence, increasing the number of parameters in the model. As more model parameters indicate higher modeling capacity, the modeling capacity needs to be regularized/restricted to avoid overfitting (i.e., identity mapping with autoencoders), as discussed in Sect. 6.2.

In addition, organizing computation through the composition of many nonlinearities helps the neural network to perform on more complex data. In other words, nonlinear activations can increase the modeling capacity. Similarly, the regularization techniques (Sect. 6) can be applied on deep overcomplete autoencoders for avoiding identity mapping.

### Deep undercomplete with space expansion

**Fig. 8:**
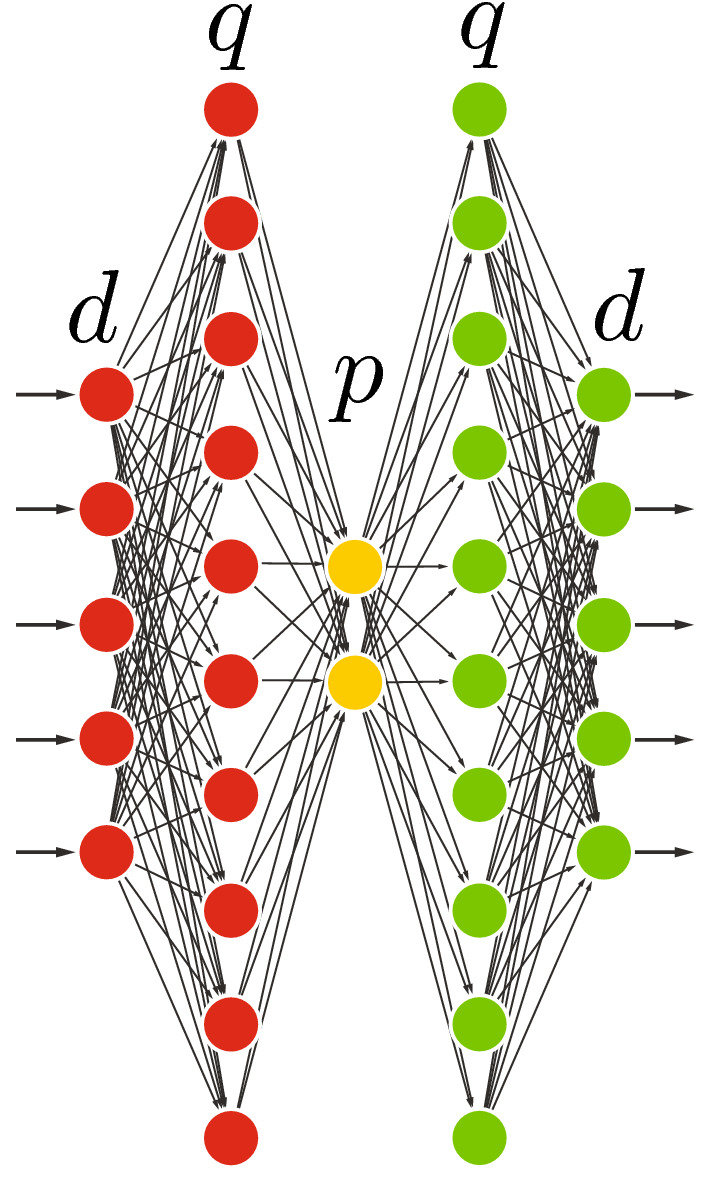
Deep undercomplete autoencoder with space expansion. This architecture is the most reminiscent of kernel-based approaches, starting with a nonlinear space expansion *F*().), also associated with a full rank *q* expansion, followed by rank-*p* reduction

As previously discussed, bottleneck layers (with or without nonlinear activation) can work as a constraint/regularizer while forcing the autoencoder to learn salient codes. However, in the spirit of SVM and other kernel-based learning methods (Vapnik 1999; Cristianini and Shawe-Taylor 2000), it may be advantageous to first expand the space in a nonlinear way before reducing the rank of the matrix through a bottleneck layer. This is what is illustrated by Fig. 8. The nonlinear expansion layer is expected to project the input features into a high-dimensional space where the relational latent factors in the input are easier to model. The compression (bottleneck) layer then extracts the salient features of the projected input data.

In spite of our experimental efforts in the context of the datasets presented in Sect. 4, we were never able to beat our shallow undercomplete autoencoder in terms of MSE and accuracy at the same time. However, as for the classification of the extracted bottleneck features, this configuration provided the highest accuracy for both MNIST digit classification and AMI IHM phone classification tasks. This observation supports the point made at the end of Sect. 3. The non-optimal MSE solutions, corresponding to local minima or to constrainted/regularized (suboptimal MSE) solutions can result in better encodings (i.e., more discriminative encodings for classification tasks), since MSE criterion is not necessarily related to classification or regression performance.

## Domain-specific autoencoders

### Context and rationale

In (Bourlard and Kamp 1988), it was shown that shallow undercomplete autoencoders with linear decoder (linear output units) and MSE as loss function learn the weight vectors that are not constrained to form an orthonormal basis, nor to have a meaningful ordering. However, it was also shown that, whether we have linear or (any) nonlinear function on the hidden layer units, the *optimal* solution spans the same subspace as the eigenvectors, and thus the aforementioned shallow undercomplete autoencoder converges to the optimal solution which is provided by PCA/SVD. As described in Sects. 6 and 8; however, there exists other autoencoder configurations.

Thanks to the alterations in their configuration as well as in their objective functions, autoencoders can be used as possible solutions to many learning problems which can be modeled as a transformation of the (input) feature space. Hence, in this section, we will further elaborate on different autoencoder architectures with respect to their domains of application.

### Feature learning

One of the earliest motivations for the autoencoders is to learn lower dimensional (i.e., bottleneck) codes (i.e., embeddings, encodings) that are efficient for not only computational but also visualization purposes. However, conventional AE with fully connected layers is not particularly designed to model all kinds of data such as two-dimensional data and sequential data.

Convolutional autoencoder (CNN AE) (Masci et al. 2011) achieves this by replacing the fully connected layers with convolutional layers in the encoder and decoder of the network. In encoding phase, a global weight matrix is used for convolution operation. The weights are shared among all locations in the input. This preserves the spatial locality, which is especially crucial while processing the images. In reconstruction phase (decoding phase), same global weight matrix is flipped over both dimensions and used for deconvolution operation. Analogous to the greedy, layer-wise pre-training with AEs for weight initialization (Bengio et al. 2007), CNN AE stack can be used to initialize CNN with identical topology prior to a supervised training stage (for image classification).

Similarly, LSTM AE (Srivastava et al. 2015) is designed for the sequential data which accommodates temporal dependence by replacing fully connected layers with LSTM units as encoder and decoder of the network inspired from Sutskever et al. (2014). LSTM AE (Srivastava et al. 2015) can be used for learning the representation of sequences of images in the unsupervised setting where there is only a dataset of unlabeled videos. In Li et al. (2015), LSTM AE is used for hierarchically building an embedding for a paragraph from embeddings for sentences and words. The decoder of the model then decodes this embedding to reconstruct the original paragraph.

### Noise reduction

Apart from encodings, we can also work with reconstructions for the enhancement of the input data as in the case of noise reduction in images (Xie et al. 2012), speech (Lu et al. 2013), and other kinds of signals (Xiong et al. 2016), usually by means of denoising autoencoders (DAE).

When using denoising autoencoder, it is also convenient to adapt the type of the layers according to the data type. For instance, CNNAE with denoising criterion can be suitable for noisy images for noise reduction (Charte et al. 2020). Similarly, an LSTM AE with denoising criterion is suitable for corrupted signals or sequences.

### Anomaly detection

Autoencoders can be simply in use for detecting the abnormal samples in data. The idea is to train autoencoder with data samples from only one class (e.g., majority class). This way the autoencoder is capable of reconstructing the input with good reconstruction loss. Thus, if data samples from another distribution are fed to the model, it is expected to result in comparatively bad reconstruction loss. Additionally, a threshold for reconstruction loss can be set to make the decision for anomaly samples.

Specifically, the ability of LSTM units for learning patterns in data over long sequences makes them appropriate design choice for anomaly detection in multivariate time series data (Nguyen et al. 2021; Principi et al. 2019).

### Robustness

Autoencoders can be used for extracting robust features from input data for the given task. Contractive AE (CAE) (Rifai et al. 2011) is one example of such models thanks to its contractive penalty term (21) which forces CAE to learn encodings which are robust to the alterations in the input data.

In addition, in Qi et al. (2014), robust stacked autoencoder (Robust AE) for dealing with data containing non-Gaussian noises and outliers is proposed. Robust AE improves upon the anti-noise ability of traditional autoencoders by replacing MSE with maximum correntropy criterion (MCC). Correntropy was initially proposed as a localized similarity measure based on information-theoretic learning (ITL) and kernel methods. Robust AEs attempt to maximize this measure (equivalently, minimize negative correntropy), which translates in a higher resilience to non-Gaussian noise.

### Data generation

As explained earlier in Sect. 6.2, autoencoders with stochastic functions (e.g., VAE) can be utilized for generating data samples that are not originally present in the training set, but still close to the samples in the training set. Thanks to these stochastic mappings, the encoder does not map an input data instance to a single point in the embedding space. Instead, it maps it to a distribution (defined by its mean and standard deviation). Hence, when reconstruction is produced by sampling from this distribution and propagating through the decoder, it does not necessarily come from an instance in the original input feature space, but still produce a coherent result with respect to the data instances in the training set.

In addition, contractive autoencoders (CAE) can also be sampled, as it can generate new instances from the learned model, by using the Jacobian of the encoder to add a small noise to another point and computing its codification (Charte et al. 2020).

## Conclusions

The main goal of this paper was primarily to revisit the conclusions of the well-cited paper (Bourlard and Kamp 1988) published in 1988 and to theoretically prove that a shallow undercomplete autoencoder, even with nonlinear functions on the hidden layer was at best, equivalent to principal component analysis (PCA) or singular value decomposition (SVD). In other words, even with a nonlinear function applied to the hidden layer, the optimal solution is given by well-understood linear algebra. In the present paper, we further validated this conclusion by experimenting on datasets which were not available at that time. We experimented on MNIST handwritten digits dataset and AMI-IHM speech dataset. With our experiments, apart from only focusing on the optimal solution in terms of reconstruction loss as in Bourlard and Kamp (1988), we also inspected the quality of the encodings (i.e., embeddings, extracted features) for digit classification and phone classification tasks, respectively.

With the hope of further improving on the performance obtained for the aforementioned tasks, both in terms of reconstruction and classification performance (by sending the extracted features to a reasonably simple DNN classifier), we also examined and experimented with more complex deep autoencoder architectures. Unfortunately, none of those models could yield significant improvements on MSE cost and only deep undercomplete autoencoders with nonlinear space expansion (reminiscent of kernel-based approaches) maintained improvements on the classification accuracy.

In addition, to reflect the advances made in autoencoder domain since 1988, we presented a brief overview of different autoencoder configurations, including shallow overcomplete autoencoders, regularized autoencoders such as sparse, contractive, denoising, variational, and adversarial autoencoders.

In conclusion, besides providing a brief overview of the advances made in autoencoder domain since 1988, we validated and even expanded the conclusions from Bourlard and Kamp (1988) with our experimental analysis and showed that (1) it remains hard (although feasible) to go beyond the PCA/SVD solution for reconstruction performance, and (2) good reconstruction performance does not necessarily mean good encodings (for classification) learned at the hidden layer(s).

## Footnotes

1 To highlight the scientific impact of our Journal over the last decades, we asked authors of highly influential papers to reflect on the history of their study, the long-term effect it had, and future perspectives of their research. We trust the reader will enjoy these first-person accounts of the history of big ideas in Biological Cybernetics.

2 Biological Cybernetics is an interdisciplinary medium for theoretical and application-oriented investigations of information processing and control in organisms. A broad goal of the Journal is to foster and intensify the search for general principles of communication and regulation in biological systems, through detailed studies of specific examples. https://www.springer.com/journal/422.

3 http://yann.lecun.com/exdb/mnist/.

4 http://yann.lecun.com/exdb/mnist/.

5 https://groups.inf.ed.ac.uk/ami/corpus/.

6 A vector of zeros except for the entry at the index representing the corresponding element (e.g., word in the vocabulary). Only that particular entry would be one.
